# A Natural Language Processing Method Identifies an Association Between Bacterial Communities in the Upper Genital Tract and Ovarian Cancer

**DOI:** 10.3390/ijms26157432

**Published:** 2025-08-01

**Authors:** Andrew Polio, Vincent Wagner, David P. Bender, Michael J. Goodheart, Jesus Gonzalez Bosquet

**Affiliations:** 1 Department of Obstetrics and Gynecology, University of Iowa, 200 Hawkins dr., Iowa City, IA 52242, USA; 2 Holden Comprehensive Cancer Center, University of Iowa Hospitals and Clinics, Iowa City, IA 52242, USA

**Keywords:** ovarian cancer, prediction model, natural language processing, microbiome, RNA sequencing, RNAseq

## Abstract

Bacterial communities within the female upper genital tract may influence the risk of ovarian cancer. In this retrospective cohort pilot study, we aim to detect different communities of bacteria between ovarian cancer and normal controls using topic modeling, a natural language processing tool. RNA was extracted and analyzed using the VITCOMIC2 pipeline. Topic modeling assessed differences in bacterial communities. Idatuning identified an optimal latent topic number and Latent Dirichlet Allocation (LDA) assessed topic differences between high-grade serous ovarian cancer (HGSOC) and controls. Results were validated using The Cancer Genome Atlas (TCGA) HGSOC dataset. A total of 801 unique taxa were identified, with 13 bacteria significantly differing between HGSOC and normal controls. LDA modeling revealed a latent topic associated with HGSOC samples, containing bacteria *Escherichia/Shigella* and *Corynebacterineae*. Pathway analysis using KEGG databases suggest differences in several biologic pathways including oocyte meiosis, aldosterone-regulated sodium reabsorption, gastric acid secretion, and long-term potentiation. These findings support the hypothesis that bacterial communities in the upper female genital tract may influence the development of HGSOC by altering the local environment, with potential functional implications between HGSOC and normal controls. However, further validation is required to confirms these associations and determine mechanistic relevance.

## 1. Introduction

The human microbiome is a symbiotic community of bacteria, fungi, and viruses that live on or within the human body with specific functions, properties, and interactions within its environment [1,2]. Bacterial communities may influence the risk of cancer within the female genital tract, specifically ovarian cancers, by altering the local microenvironment. Alterations within the microbiome may lead to inflammation, immune response modulation, genetic or epigenetic changes, or microenvironment modulation, which can lead to the development of gynecologic cancers [3,4,5]. Historically, identification within the microbiome relied on culture studies, however, 16S RNA gene sequencing is now the test of choice. It is a culture-independent method that utilizes the highly conserved 16S RNA gene to evaluate bacterial diversity from complex microbiomes [6,7]. Topic modeling, a natural language processing tool, can be used to assess latent interactions between microbes, providing a representation of the community of bacteria related to various states. Along with functional analysis, this allows unique insight bacterial community structure associated with healthy or disease states [8].

The objective of this pilot study was to detect differences in the bacteria of the upper genital tract in high-grade serous ovarian cancer (HGSOC) and normal control samples using 16S RNA sequencing and topic modeling. Results were externally validated in The Cancer Genome Atlas (TCGA) HGSOC dataset. Pathway analysis was then performed using the Kyoto Encyclopedia of Genes and Genomes (KEGG) database.

## 2. Results

**Figure 1:**
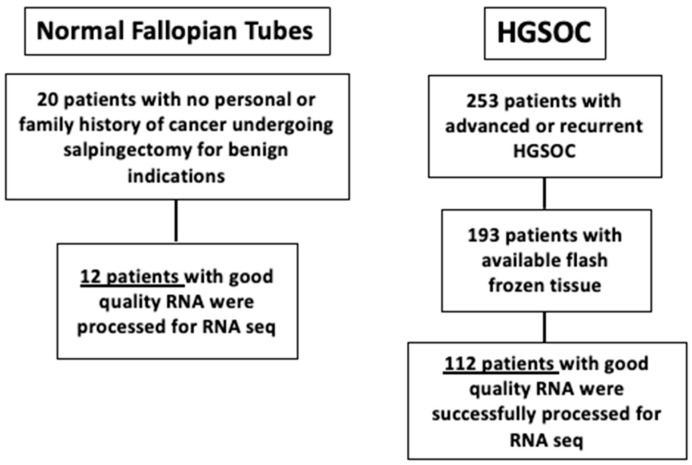
Patient population. Normal fallopian tube samples from patients with no risk factors and no personal/family history of ovarian cancer. Out of the 20 samples, 12 were suitable and were sequenced. Samples were from HGSOC patients that underwent surgical intervention at the University of Iowa Hospitals and Clinics and had their tumors sequenced.

A total of 253 patients with advanced or recurrent HGSOC were identified, with 193 patients with available tissue. Ultimately, 112 patients had tissue samples with good quality RNA and were processed for RNA sequencing. Then, 20 patients undergoing salpingectomy for benign indications were identified, and 12 patient samples were ultimately identified to have good quality RNA which was processed for RNA sequencing (Figure 1).

**Figure 2:**
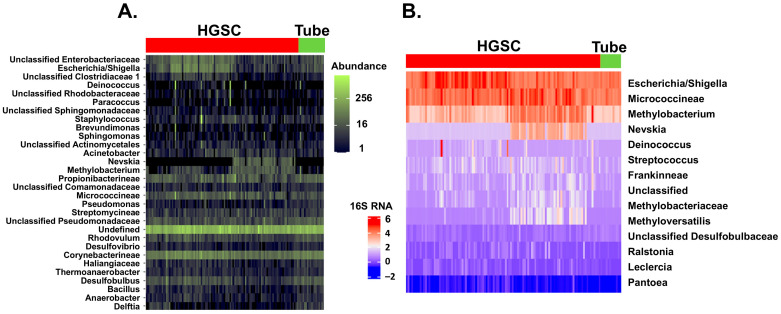
Comparison of 16S RNA gene expression between HGSOC and control samples. (**A**) Heatmap of the normalized 30 most frequent bacterial 16S RNA counts found by RNAseq for HGSOC and control samples. Abundance counts are represented in green. Analysis was performed by phyloseq R package (v 4.4.1). (**B**) Heatmap of the 16S RNA log−2 transformed normalized counts found by RNAseq between HGSOC and control samples that were different following univariate analysis, N = 13. Analysis was performed with DESeq2 R package. 16S RNAlog2 transformed expression is represented in a blue–red scale.

Following 16S RNA analysis, a total of 801 unique bacterial taxa were identified in the 124 samples. The univariate analysis highlighted thirteen taxa significantly different in relative abundance counts between HGSOC and control samples (*p* < 0.05) (Figure 2).

**Figure 3:**
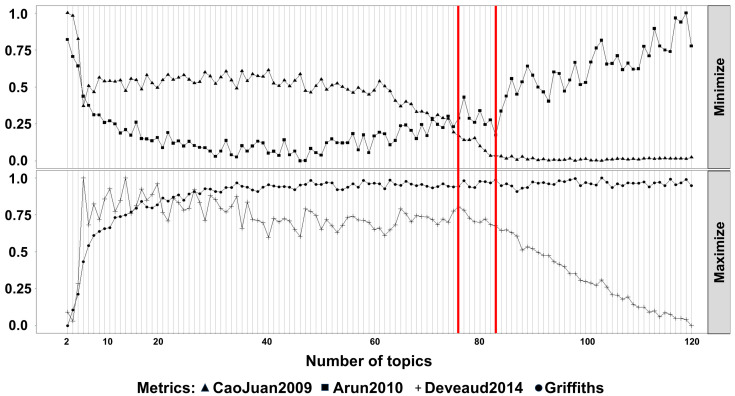
Analysis to identify an optimal latent of topic numbers in the cohort. The *FindTopicNumber* function from the Idatuning (v 1.0.3) package was used to identify an optimal latent number topic using both minimization (CoaJuan2009, Arun2010) and maximalization (Griffiths, Deveaud2014) metrics. Based on these metrics #83 was selected as the model to proceed. On the horizontal axis, number of topics tested, from 0 to 120. On the vertical axis the percentage of variation.

**Figure 4:**
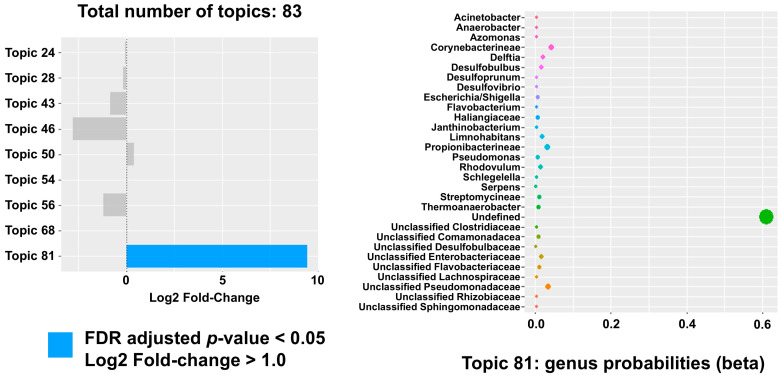
Topic modeling using Latent Dirichlet Allocation (LDA). LDA is a natural language processing tool for topic modeling that assesses for differentially abundant topics between HGSOC and control samples. Left panel: topic #81 demonstrates positive log2 fold changes (>1) with over 9-fold change between cancer and control samples, and with a significant FDR-adjusted *p*-value (*p* < 0.05). Right panel: plotting per-topic bacterial (vertical axis) probabilities (horizontal axis).

LDA modeling was applied to discern latent topics within the bacterial community data. Using the Idatuning method, the optimal number of latent topics was identified as 83. Topic #81 was identified as significantly different in HGSOC samples with a positive log2 fold change (FDR-adjusted *p* < 0.05). This topic included bacteria like *Escherichia/Shigella*, *Corynebacterineae* (Figure 3 and Figure 4). To validate these findings, a similar analysis was performed using data from the TCGA dataset. In total, 868 unique taxa were identified with 43 optimal latent topics. Topics #19 and #36 were found to be significant, which included 33% of the genera observed in the initial significant topic, including *Escherichia/Shigella* and *Corynebacterineae*.

**Figure 5:**
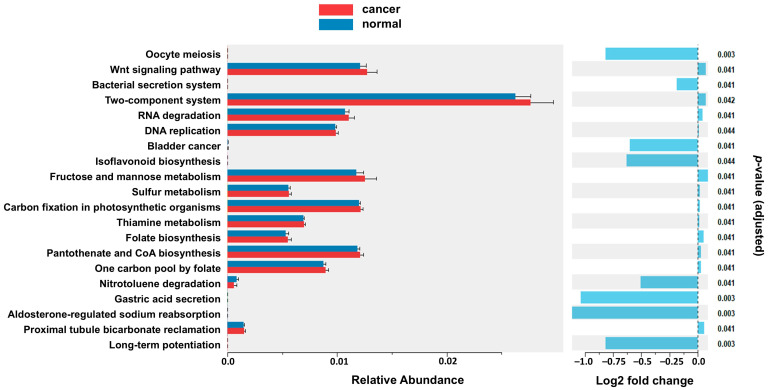
KEGG pathway differences between HGSOC and normal controls. Multiple signaling pathways are found to be significantly different including environmental information processing, metabolic, and organismal systems. Mid-panel vertical axis: KEGG name of the significant pathways; lower axis: relative gene expression abundance (log2 transformed) in the described pathways. Right panel: log2 fold change with direction; negative: less in cancer than in normal; positive: more in cancer than in normal. Right-side, adjusted *p*-value of the difference.

After predicting pathway abundance with *picrust2* (v 2.6.2), we performed differential pathway analysis between HGSOC samples and controls using KEGG pathways database identifying enrichment of multiple signaling pathways including oocyte meiosis, aldosterone regulated sodium reabsorption, gastric acid secretion, and long-term potentiation (negative log2 fold change < 1, *p* = 0.003) (Figure 5).

**Figure 6:**
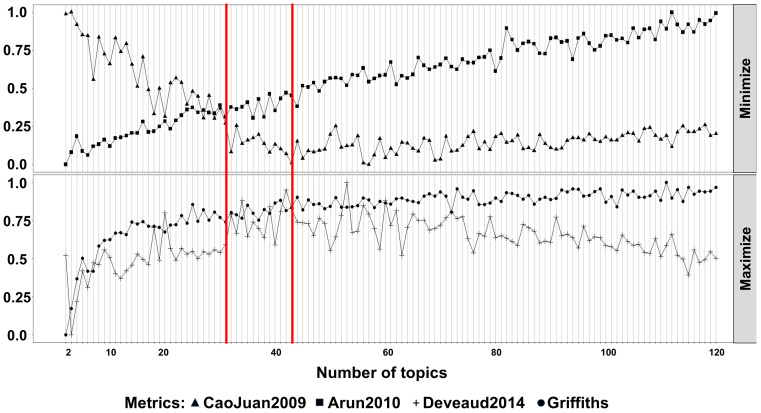
Analysis to identify an optimal latent of topic numbers in the TCGA cohort. The *FindTopicNumber* function from the Idatuning (v 1.0.3) package was used to identify an optimal latent number topic using both minimization (CoaJuan2009, Arun2010) and maximalization (Griffiths, Deveaud2014) metrics. Based on these metrics #43 was selected as the optimal topic number to proceed. On the horizontal axis is the number of topics tested, from 0 to 120. On the vertical axis is the percentage of variation.

The results were then externally validated using the TCGA HGSOC dataset. A similar analysis was performed which identified 868 unique taxa. Topic modeling was then again utilized to analyze changes in bacterial communities between the two sample groups (Figure 6). An optimal latent topic number was identified as 43.

**Figure 7:**
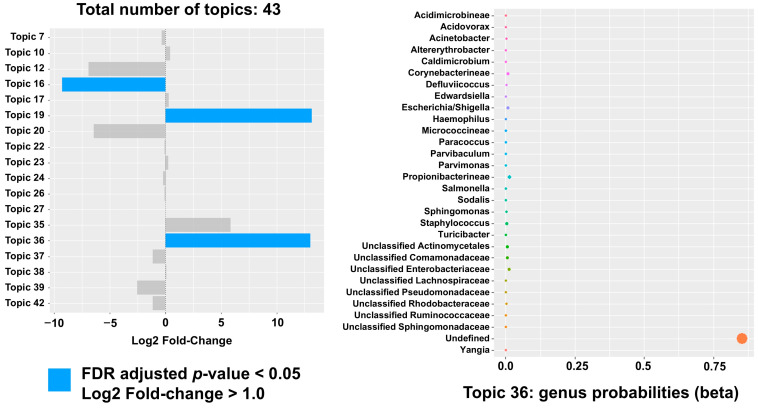
Topic modeling using Latent Dirichlet Allocation (LDA). LDA topic modeling in the TCGA dataset. Left panel: topics #19 and #36 demonstrate positive log2 fold changes (>1), and topic #16 demonstrates negative log2 fold changes between cancer and control samples. All three have significant FDR-adjusted *p*-values (*p* < 0.05). Right panel: plotting bacterial (vertical axis) probabilities (horizontal axis) of topic #36.

Once identified, LDA was used to identify several significantly different topics including topics #19 and #36. These topics included 33% of the same genera observed in the study significant topic #81 including *Escherichia/Shigella*, *Corynebacterineae* (Figure 7).

## 3. Discussion

The current study presents a novel application of natural language processing (NLP) through topic modeling to identify potentially significant differences in bacterial communities in the upper genital tract of patients with HGSOC. Our findings revealed differences in bacterial communities between HGSOC and normal control samples which may reflect underlying dysbiosis, however, these associations remain correlative. Among these, genera such as *Escherichia/Shigella* and members of the *Corynebacterineae* family were notably associated with HGSOC, consistent with the previous literature suggesting that alterations in the microbiome may contribute to disease pathogenesis [9,10]. The enrichment of genera such as *Escherichia/Shigella* and *Corynebacterineae* in HGSOC samples aligns with prior studies that have reported their presence in ovarian cancer or peritoneal environments [3,4,9,10]. Emerging evidence suggests microbiome communities within the upper female genital tract may influence the development and/or progression of ovarian cancer. While causality remains elusive, several studies have reported associations between microbiome dysbiosis, inflammation, and tumorigenesis. Microbiome involvement in the initiation and progression of cancer can be mediated by modulation of the immune system. *Escherichia/Shigella*, for example, are Gram-negative bacteria that can activate Toll-like receptor 4 (TLR4) promoting inflammation and activation of the NF-kB pathway, producing pro- and anti-inflammatory cytokines. The bacterial metabolite, lipopolysaccharide (LPS), is highly immunogenic and can stimulate tumor progression through TLR4, which subsequently induces phosphatidylinositol-3-kinase (PIK3) and activation of EMT [11,12,13]. Microbial dysgenesis and overactivation of these pathways may promote a cancer permissive environment [14].

Furthermore, the enrichment of specific taxa may exert biologic influence through hormonal modulation. One such mechanism is via microbially mediated changes in estrogen metabolism. Prior work has shown certain gut and reproductive tract microbes possess β-glucuronidase activity which may modulate systemic estrogen levels by reactivating conjugated estrogens in the enterohepatic, potentially leading to elevated local hormonal concentrations in the pelvic environment. Altered estrogen signaling can promote proliferation, DNA damage, and impair apoptosis in ovarian tissue, contributing to ovarian carcinogenesis [15,16,17]. In our previous work, we observed that certain bacterial taxa were associated with patterns of somatic DNA methylation and genomic alterations in HGSOC [18], suggesting that microbiota may shape tumor evolution by influencing host epigenetic and transcriptional programs [18]. Taken together, these may support the hypothesis that microbial communities may contribute to the composition and evolution of the tumor microenvironment.

However, bacterial function often relies on, or is influenced by, other members within the specific community [19]. Using topic modeling, it is possible to deconstruct complex microbial communities into a set of topics that represent the distribution of full communities [20]. LDA modeling identified topic #81 as the optimal latent topic which showed a positive fold change in HGSOC samples compared to controls, indicating an overrepresentation of specific bacterial taxa in cancerous tissues. This supports the hypothesis that the composition of a specific bacterial community may play an integral role in the development of disease. This topic primarily included bacterial species known to reside in the genital tract, reinforcing the hypothesis local microbial dysbiosis influences ovarian cancer development.

The use of NLP and topic modeling offers a unique perspective on microbial analysis. It allows for potential investigation into not only how communities differ quantifiably, but also in their composition. By treating bacterial communities as “topics” like how words cluster in textual data, we were able to model the high-dimensional interactions between different bacterial species and identify potentially meaningful patterns and associations with ovarian cancer.

Additionally, our pathway analysis revealed potential alterations in several biologic processes between samples. Oocyte meiosis and aldosterone-regulated sodium reabsorption pathways are relevant to ovarian function and were noted to be downregulated in HGSOC samples [21]. Disruptions in oocyte meiosis can affect cell division, chromosomal integrity, and repair mechanisms resulting in accumulation of mutations and promotion of cancer development. Alterations within the aldosterone pathway can affect sodium and other electrolyte homeostasis, fluid retention affecting the local tumor microenvironment. The affected pathways of oocyte meiosis and aldosterone regulated sodium reabsorption may reflect microbial influence on host signaling via microbial derived metabolites. For example, microbial derived short chain fatty acids (SCFAs) can modulate histone deacetylase activity and microbiota can impact estrogen metabolism; both relevant to ovarian physiology and tumorigenesis [22]. Changes within systemic metabolic and organismal systems, including gastric acid secretion and long-term potentiation, suggest the potential for broader effects resulting from local microbiome imbalances within the genital tract. While not mechanistically definitive, taken in full, this poses the hypothesis that these may reflect broader metabolic disruption that may affect immune response, cell signaling pathways, and the local tumor environment, contributing to the development and growth of cancers within the genital tract.

A strength of this study is the integration of multiple analytic tools, which allowed us to identify differences in composition and a potential understanding of functional implications of these microbial shifts. Validation of our results with the TCGA dataset also adds robustness to our findings and external validity. The limitations of this study include its retrospective nature. This limits our ability to establish causal relationships between microbial differences and the development of disease. There is the potential for confounding factors, such as patient demographics, antibiotic use, or other factors that may contribute to changes within the microbiome. Discrepancies between datasets may be due to a variability in sample acquisition, sequencing depth, or RNA extraction methods. PICRUSt2 predictions are limited by reliance on reference genomes and 16S rRNA data. Future studies using shotgun metagenomics will be essential to validate functional predictions and explore strain-specific contributions. Additionally, while results were validated using an independent dataset, TCGA, the study did not employ independent analytical platforms to validate and confirm microbial signal. Therefore, these findings could potentially be subject to contamination and should be interpreted as hypothesis generating, pending further validation. Additional studies which follow microbiome changes over time, as well as clinical outcomes, would help establish a more causal role in the development of cancer. Finally, the use of topic modeling carries its own limitations, including a susceptibility to overfitting, especially with smaller sample sizes. Although the sample size, particularly for the control group was small, we accounted for this through validation with the TCGA dataset, but future efforts will include validating these findings with larger datasets.

## 4. Materials and Methods

We performed a single institution, retrospective, cohort pilot study comparing abundance of bacterial presence between HGSOC and control samples. Tumor samples were collected from patients with HGSOC undergoing cytoreductive surgery (cases) and compared to patient samples collected at the time of surgery for benign indications (controls).

### 4.1. Specimen Acquisition

Tissue samples were obtained from the Department of Obstetrics and Gynecology Gynecologic Oncology Bank (IRB, ID#200209010), which is part of the Women’s Health Tissue Repository (WHTR, IRB, ID#201809807). A separate approval was given by the University of Iowa (UI) Institutional Review Board (IRB, ID#201202714) to collect 20 normal fallopian tube samples in coordination with the University of Iowa Tissue Procurement Core Facility to be used as controls. Tubal samples came from the junction of the ampullary and fimbriated end of fallopian tubes of volunteers without any family or personal history of cancers who were scheduled to undergo salpingectomy for benign indications (mainly sterilization). Fallopian tubes were chosen as controls as current understanding is this the likely origin of HGSOC [23]. No patient indicating a personal or family history of cancer was included. All tissues archived in the WHTR were originally obtained from adult patients under informed consent in accordance with University of Iowa IRB guidelines. RNA was then extracted from epithelial tissue from the junction of the ampullary and fimbriated end of fallopian tubes. Twenty normal fallopian tube specimens were obtained. Of those, 12 produced viable RNA for analysis. RNA from both the fallopian tube and HGSC specimens had been previously extracted and purified in a prior study [18].

### 4.2. RNA Sequencing and Metagenomic Analysis

RNA was extracted and processed using the VITCOMIC2 (v 3.0) pipeline, which analyzes the 16S RNA gene and high throughput sequences to visualize the phylogenetic composition of metagenomic samples. Files from sequencing were pre-processed with *fastp* (v 0.23.4) and then *seqkit* (v 2.3.0) was applied to convert FASTQ to FASTA format [24,25]. Finally, *MAPseq* (v 2.0.1alpha) was used to map sequences against reference 16S RNA sequences. This also provides a curated reference of full-length rRNA genes and pre-classified to taxonomic categories based on the NCBI taxonomy and All-species Living Tree Project dataset. We used hits with an identity >94% and an alignment length of ≥75 base pairs. These parameters helped exclude likely contaminant sequences and human RNA, ensuring a high-confidence microbial profile. Host depletion was effectively achieved by focusing on reads mapping specifically to conserved 6S rRNA genes. VITCOMIC2 (v 3.0) was then used to determine the bacterial composition of the samples [26,27].

The *Phyloseq* package (v 1.52.0) was used for the representation and analysis of microbiome census data. *DESeq2* (v 1.49.3) was used to normalize, log2 transform, and analyze count data. A univariate analysis identified differences in relative bacterial 16S RNA abundance counts. All samples were processed using standardized extraction, handling, and sequencing pipelines at a single institution to minimize batch effects or technical confounding.

### 4.3. Natural Language Processing Analysis

Topic modeling with Latent Dirichlet Allocation (LDA) was used to assess changes in bacterial communities between samples: (1) first, the Idatuning method determined the optimal number of latent topics for the analysis. This tool is commonly used in NLP to optimize topic models like LDA. Idatuning systematically tests multiple topic counts and applies statistical metrics to identify the best-fitting model. (2) Then, we used Topicmodels to evaluate differences in bacterial communities by examining topic distributions. Statistical differences between the two groups (HGSOC vs. controls) were considered for false discovery rate (FDR)-adjusted *p*-values < 0.05. A schematic of this pipeline is available in the Supplementary Materials.

### 4.4. Prediction of Functional Profiles

Functional profiles cannot be directly identified using 16S rRNA gene sequence data, so several methods have been developed to predict microbial community functions from taxonomic profiles. One of them is picrust2 (Phylogenetic Investigation of Communities by Reconstruction of Unobserved States, v 2.6.2), which was developed for prediction of functions from 16S marker sequences [28]. Functional prediction was based on gene families resulting from the KEGG orthologs and Enzyme Classification numbers (EC) datasets analyses. With picrust2 we predicted the pathway abundance, and then with the R package ggpicrust2, we performed the differential pathway abundance between cases and controls [29].

### 4.5. Analysis Validation

Validation with TCGA database: Validation was performed using HGSOC TCGA dataset. Briefly: after permission was granted to access controlled data by the Genomic Data Commons (GDC) Data Portal (dbGaP#29868), TCGA HGSOC RNAseq (N = 423) files in BAM format were downloaded from women with HGSOC. As previously described, we used the VITCOMIC2 pipeline to assess the phylogenetic composition of metagenomic samples. Briefly, BAM files were converted to FASTQ format with *samtools*. Then, FASTQ were pre-processed with *fastp* and then *seqkit* was applied to convert to FASTA format. Finally, *MAPseq* was used to map sequences against reference 16S RNA sequences with a curated reference of taxonomic categories. We used hits with identity >94% and an alignment length of ≥75 base pairs. We use VITCOMIC2 to determine the bacterial composition of the samples, *Phyloseq* for representation and analysis of microbiome census data, and *DESeq2* to normalize, log2 transform, and analyze count data.

## 5. Conclusions

In conclusion, NLP methods identified different bacterial communities in the upper female genital tract associated with HGSOC compared to normal controls. Differences in bacterial communities may be related to functional differences between HGSOC samples and normal controls. While this provides preliminary evidence suggesting potential association, this is hypothesis generating and further study is needed to confirm these associations and the role of the microbiome in the development of ovarian cancer. These results should therefore be interpreted as exploratory. Further studies should include larger sample sizes to validate these results, include mechanistic investigations, longitudinal tracking of microbiome changes, and investigate potential biomarkers for early detection in ovarian cancer.

## Data Availability

Clinical data are not publicly available due to patient privacy. Datasets can be browsed by their accession number: GSE156699. The validation part of this study was performed in silico, with de-identified publicly available data. All data from TCGA are available at their website: https://portal.gdc.cancer.gov/, accessed 20 December 2024. Software utilized by this study is also publicly available at Bioconductor website: http://bioconductor.org/, accessed 20 December 2024.

## Supplementary Materials

The following supporting information can be downloaded at: https://www.mdpi.com/article/10.3390/ijms26157432/s1.
