# Supplementary figures and images for: A Natural Language Processing Method Identifies an Association Between Bacterial Communities in the Upper Genital Tract and Ovarian Cancer
Source: Int J Mol Sci. 2025 Aug 1;26(15):7432. doi: 10.3390/ijms26157432 (PMC12347966; doi:10.3390/ijms26157432)

# Metagenomics Pipeline

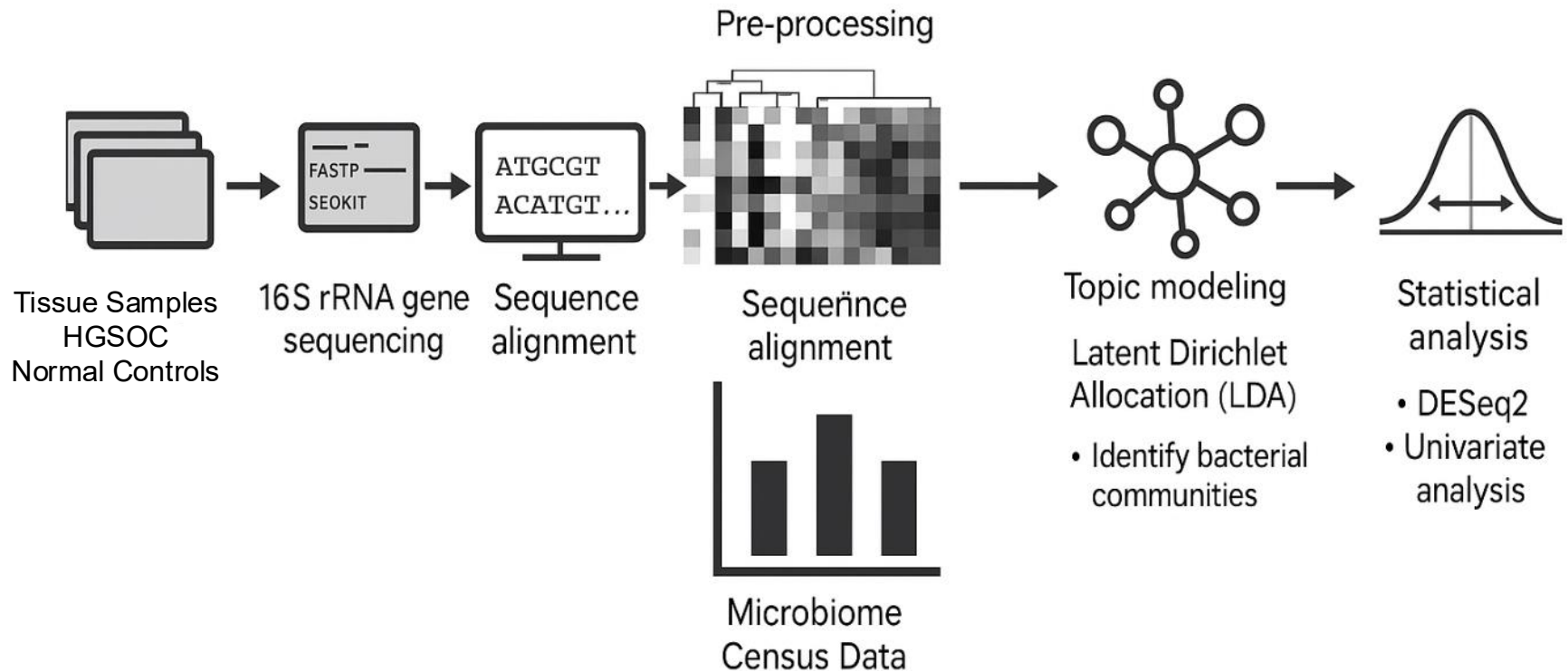

Supplement: Supplementary file 1 [file ijms-26-07432-s001.zip › 16S_rRNA_Topic_Modeling_Schematic_Complete.pdf]
